# Revealing the effects of maternal di(2‐ethylhexyl) phthalate exposure on the progression of early meiosis in female foetal germ cells at single‐cell resolution

**DOI:** 10.1002/ctm2.687

**Published:** 2022-04-07

**Authors:** Zi‐Hui Yan, Lu Wang, Wei Ge, Hai‐Xia Liu, Tao‐Ran He, Yan‐Qin Feng, Ming‐Hao Li, Jun‐Jie Wang, Ai‐Hong Zhao, Wei Shen

**Affiliations:** ^1^ College of Life Sciences Key Laboratory of Animal Reproduction and Biotechnology in Universities of Shandong Qingdao Agricultural University Qingdao China; ^2^ Qingdao Academy of Agricultural Sciences Qingdao China

Dear Editor

To provide in‐depth insight into how di(2‐ethylhexyl) phthalate (DEHP) influences early oogenesis, this study outlined the effects of maternal DEHP exposure on the meiotic progression of female germ cells in mice foetuses in vivo. DEHP is widely distributed in our lives owing to the daily use of polyvinyl chloride in plastic products.^1^, ^2^ Multiple reports have demonstrated that DEHP intake is particularly harmful to pregnant women as there is strong evidence that DEHP can pass through the maternal–foetal barrier.^3^, ^4^ However, the exact effect of DEHP on meiotic initiation of foetal germ cells, a critical biological event for successful oogenesis during pregnancy, is not fully understood.^5^ In this research, we performed single‐cell RNA sequencing (scRNA‐seq) to analyse the foetal ovaries at 12.5 days post coitum (dpc) and 14.5 dpc after maternal DEHP exposure.

Normal saline or 40 μg/kg body weight DEHP were orally administrated to pregnant mice daily from 6.5 dpc, and 12.5 dpc or 14.5 dpc foetal ovaries were isolated for scRNA‐seq (Figure 1A). A total of 25 270 high‐quality cells were selected for further analysis (Figure S1A,B). After dimensionality reduction using uniform manifold approximation and projection (UMAP), these cells were divided into 18 clusters and seven types of cells were identified according to their canonical marker expression, including germ cells, granulosa cells, mesothelial cells, interstitial cells, endothelial cells, erythroid cells, and immune cells (Figure 1B–D; Figure S1C).^6^

**FIGURE 1:**
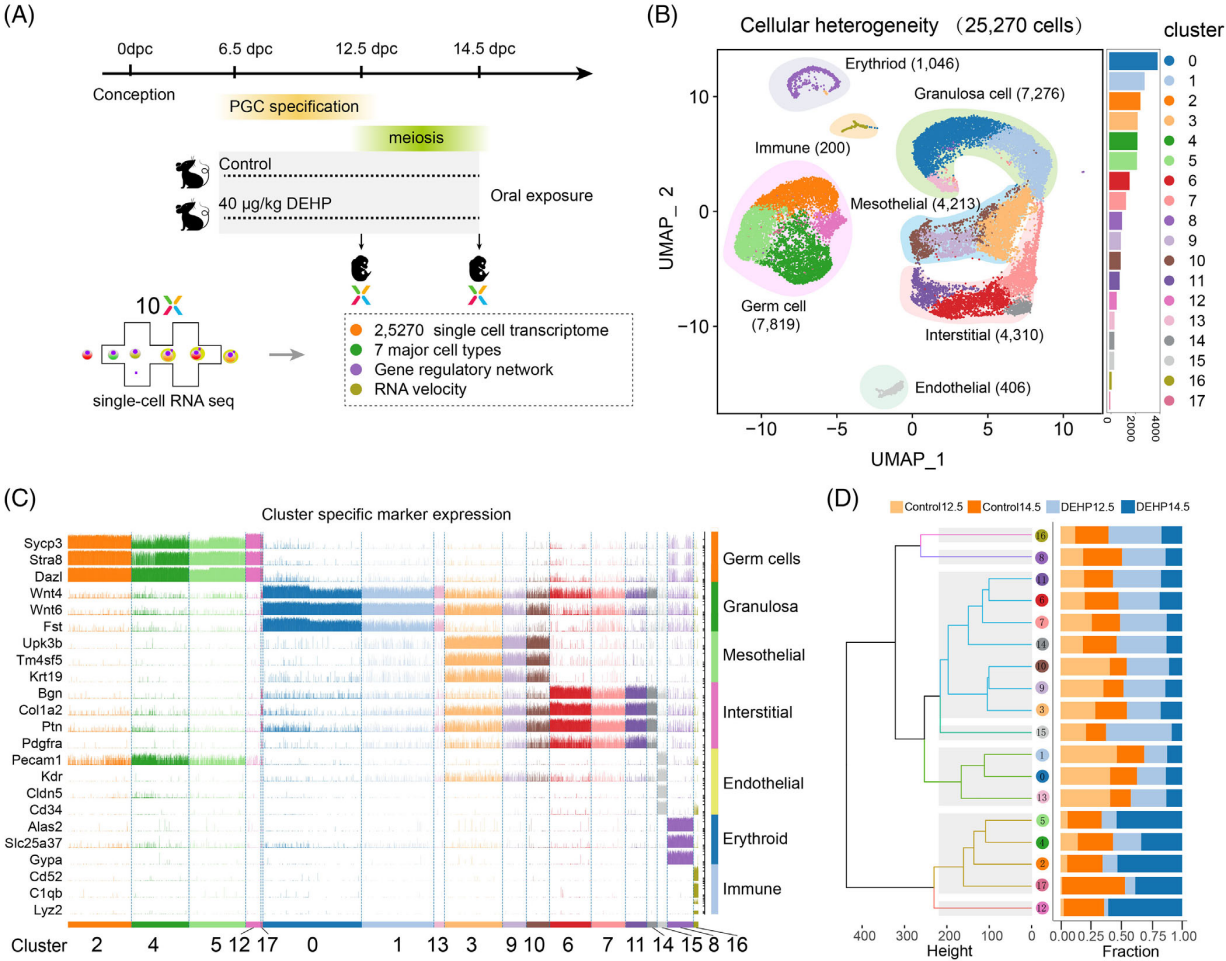
Experimental profile and gonadal ridge cell analysis. (A) Schematic diagram of DEHP treatment and the scRNA‐seq process. (B) UMAP plot of the identified gonadal cells (left) and cell numbers in each cluster (right). (C) Tracksplot of the expression of special marker genes in each cell type. (D) Dendrogram of the clusters (left) and percentages of cells from different samples in each cluster (right)

To provide a fine‐scale analysis of how maternal DEHP exposure affects the meiotic transcriptome, germ cell clusters were extracted for further analysis (Figure 2A–D). After UMAP analysis, germ cells were subdivided into 16 transcriptionally distinct clusters whose expression patterns, from left to right in the UMAP plot, correspond to pre‐meiosis, pre‐leptotene, leptotene, zygotene and pachytene stage cells (Figure 2E; Figure S2A).^7^ Noteworthy, cluster 13 showed similar expression to leptotene cells while they contained more than 80% of cells from the DEHP‐treated group (Figure 2D; Figure S2B–D). Taken together with its aberrant distribution in the UMAP plot, we, therefore, designated them as abnormal leptotene cell clusters induced by maternal DEHP exposure, which was supported by the higher proportion of abnormal leptotene cells to overall leptotene cells in the DEHP group (Figure S2E). To verify the developmental trajectory, we then performed RNA velocity analysis, an elaborate method of inferring cell fate decision based on the ratio of spliced and unspliced mRNA,^8^ the results also confirmed that both leptotene cells and abnormal leptotene cells were differentiated from pre‐meiotic cells (Figure 2F; Figure S2F), therefore confirmed our hypothesis. Together, our analysis here demonstrated that maternal DEHP exposure induced abnormal differentiation of early meiotic germ cells.

**FIGURE 2:**
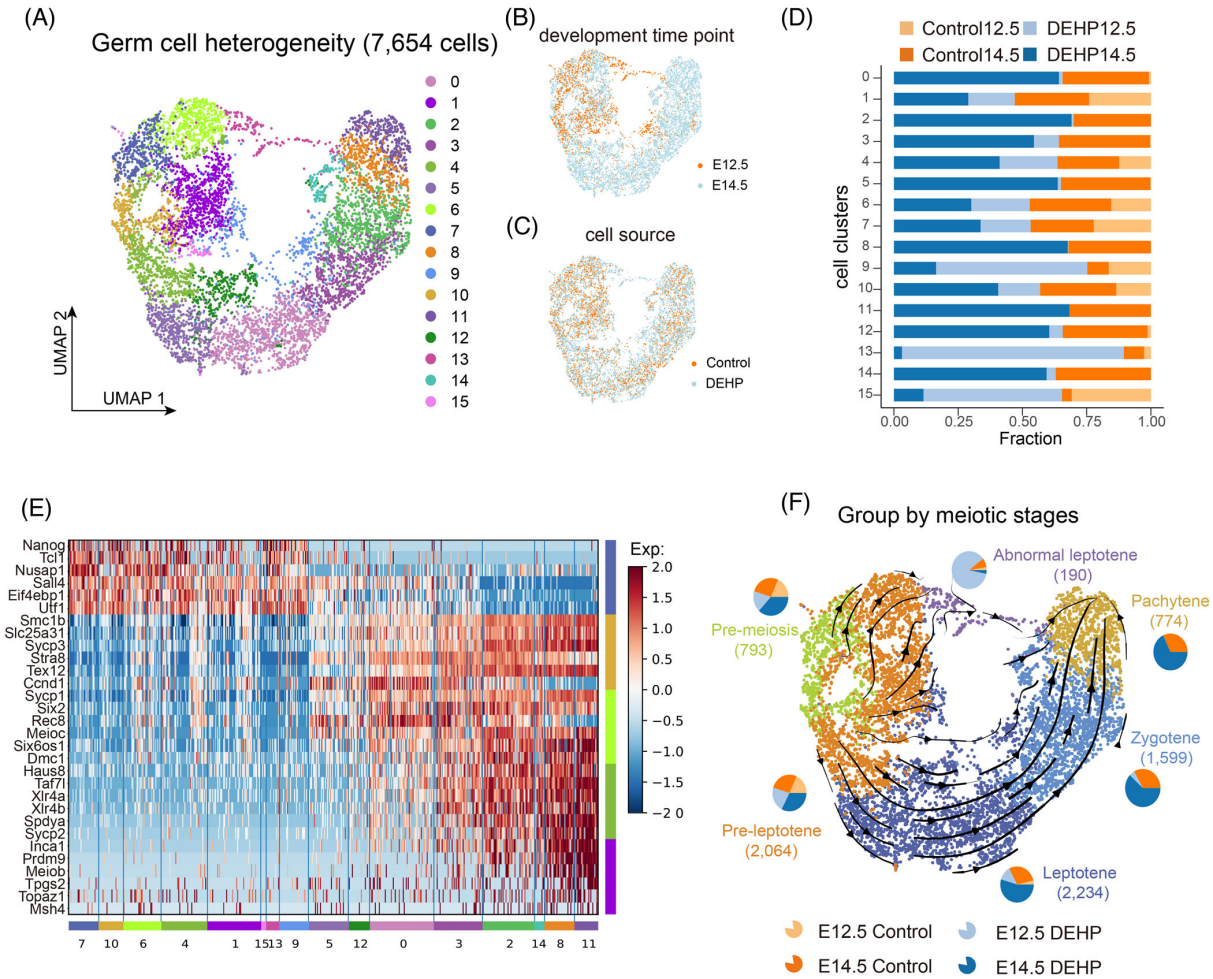
Detailed analysis and identification of germ cells. (A–C) UMAP plot of germ cells coloured by clusters (A), time points (B) and different treatments (C). (D) Percentages of germ cells from different samples in each cluster. (E) Heatmap of the expression of special marker genes in the different germ cell developmental stages. (F) RNA velocity streamlines embedded on the UMAP plot coloured by cell stage. Pie plots indicate the percentage of cells from different samples in each stage

To determine how DEHP exposure affected the abnormal developmental trajectory in female germ cells, we then performed a differentially expressed gene (DEG) analysis between leptotene and abnormal leptotene cells (Figure 3A; Figure S3A). Gene ontology (GO) analysis indicated that DNA damage‐related genes were altered after maternal DEHP exposure (Figure 3B). Further expression comparison and dynamic velocity analysis of genes correlated with meiosis, DNA damage response and apoptosis indicated that abnormal leptotene cells suffered DNA damage, failed to start normal meiosis and might undergo apoptosis (Figure S3B–D). We further performed chromosome spread analysis to evaluate DNA damage marker RAD51 and BRCA1 expression in meiotic germ cells. It was found that the percentage of RAD51‐positive leptotene cells in the DEHP group was significantly higher compared with the control group (Figure 3C). Meanwhile, the percentage of BRCA1‐positive leptotene cells was not significantly affected (Figure 3D).

**FIGURE 3:**
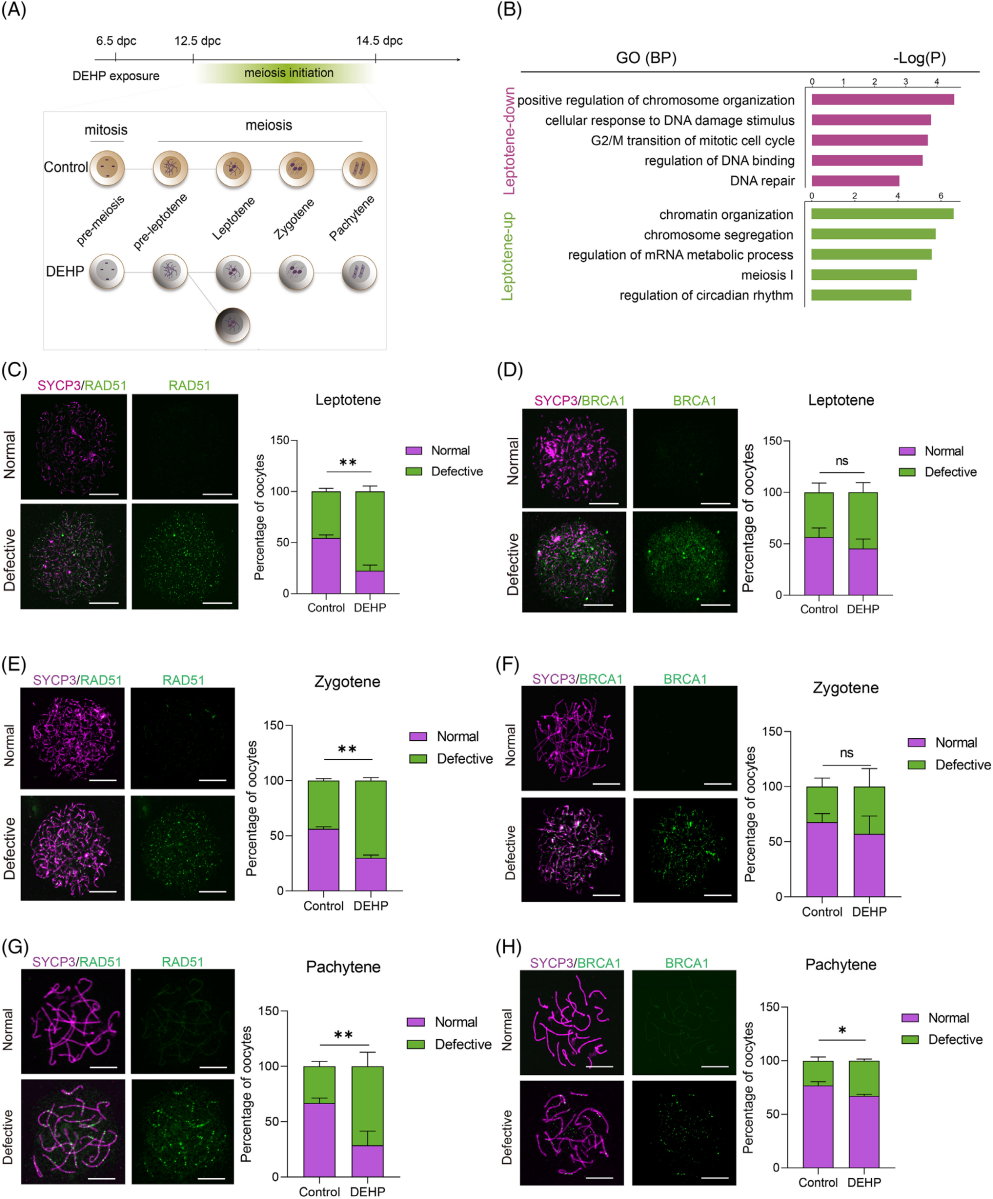
Leptotene, zygotene and pachytene germ cells were impaired by DEHP. (A) Diagram of the identified developmental stages of the control and DEHP‐treated groups. The dark grey cells with the distinct branch indicate the identified abnormal leptotene cells. (B) GO term enrichment analysis of downregulated (top) and upregulated (bottom) DEGs in leptotene stage cells compared with abnormal leptotene cells. (C–H) Chromosome spread results of leptotene (C,D), zygotene (E,F), pachytene (G,H) cells labelled by RAD51 (C,E,G) or BRCA1 (D,F,H), and the percentages of cells with normal and defective expressions of RAD51 or BRCA1 in the control and DEHP‐treated groups. Scale bar = 5 μm. The data are presented as mean ± SD. * and ** represent *p* < .05 and *p* < .01, respectively, and ns represents not significant. No less than three replicates were conducted in each experiment

After demonstrating how DEHP affected early meiotic progression, we then focused on zygotene and pachytene cells. Noteworthy, the DEGs in the two stages were similar (Figure S4A,B) and GO enrichment analysis showed that DEGs in these two stages were related to DNA damage, chromosome organisation and mitochondrial function, indicating that maternal DEHP exposure caused similar effects on these two stages (Figure S4C,D). Comparison of the representative gene expression also confirmed our GO analysis (Figure S4E). Besides, chromosome spread analysis showed that the percentages of RAD51‐positive cells after DEHP treatment were significantly higher in both zygotene and pachytene cells (Figure 3E,G). Moreover, the percentages of BRCA1‐positive cells were not significantly perturbed in zygotene cells, but significantly different in pachytene cells (Figure 3F,H).

Furthermore, as multiple reports demonstrated that mitochondrial damage correlates with DNA damage^9^ and maternal DEHP exposure perturbed substantial mitochondria functional genes in the current study, levels of mitochondria were also detected. The fluorescence intensity of the mitochondrial marker TOMM20 in germ cell cytoplasm was significantly decreased after DEHP treatment (Figure 4A,B), which was also consistent with the functional gene network interaction analysis (Figure S4F,G). Furthermore, we found that AMP‐activated protein kinase (AMPK), which might be activated due to DEHP (Figure S5A,B), was phosphorylated in more germ cells compared with the control group (Figure 4C). We further detected both the upstream and downstream proteins of AMPK and discovered that the intensity of both phosphorylated ATM (upstream) and phosphorylated ULK1 (downstream) was significantly increased after DEHP exposure, which was also consistent with our scRNA‐seq analysis (Figure 4D,E; Figure S5C,D). Taken together, we concluded that maternal DEHP exposure impaired both the DNA integrity and mitochondria function of some female germ cells. It is therefore plausible that the impaired DNA recruited ATM in response to DEHP, and the activated ATM phosphorylated AMPK. Then AMPK, as a regulator of mitochondrial homeostasis,^10^ began to phosphorylate ULK1 and clear damaged mitochondria (Figure S6).

**FIGURE 4:**
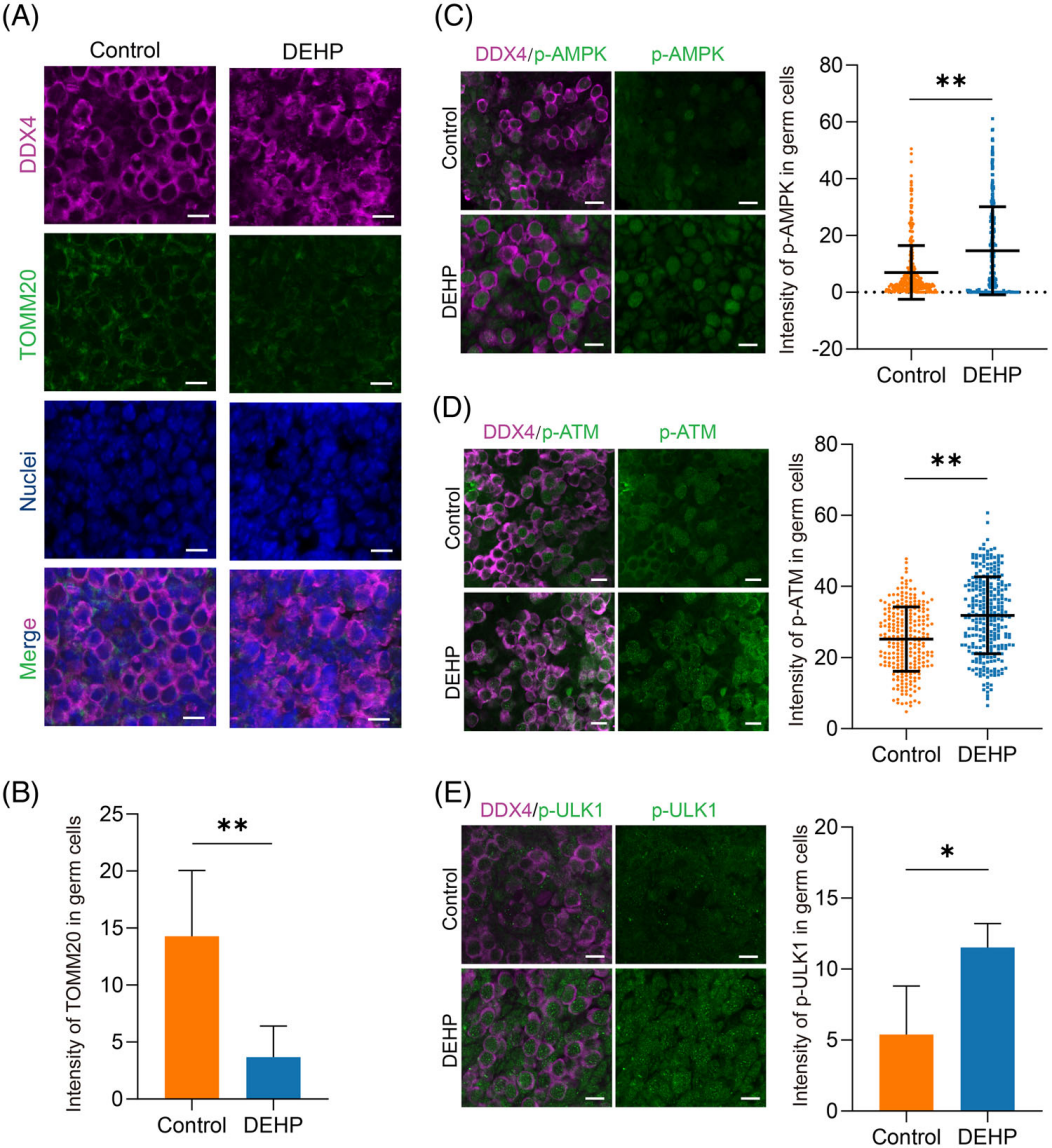
AMPK might regulate mitochondria after DNA impairment induced by DEHP. (A) Immunofluorescence plot of germ cells between the control and DEHP‐treated group. The gonadal ridges were labelled by DDX4 (purple) and TOMM20 (green). (B) The average intensity of TOMM20 between the control and DEHP‐treated group. (C–E) Immunofluorescence plot of germ cells labelled by p‐AMPK (C), p‐ATM (D), and p‐ULK1 (E), respectively, and average intensity of p‐AMPK, p‐ATM, and p‐ULK1 between the control and DEHP‐treated group. Scale bar = 10 μm. The data are presented as mean ± SD. * and ** represent *p* < .05 and *p* < .01, respectively. No less than three replicates were conducted in each experiment

In conclusion, we utilised scRNA‐seq to demonstrate the two developmental trajectories of germ cells after DEHP exposure and identified the AMPK pathway as a route by which DEHP impaired germ cells. This pathway should now be targeted for further research into the clinical prevention and treatment of DEHP exposure. The results of this paper suggest that DEHP could impair germ cell development in foetuses through maternal exposure, which therefore warrants careful use of plastic products during pregnancy.

## CONFLICT OF INTEREST

The authors declare no conflict of interest.

## Supporting information



Supporting Information
