# Supplementary material for: Revealing the effects of maternal di(2‐ethylhexyl) phthalate exposure on the progression of early meiosis in female foetal germ cells at single‐cell resolution
Source: Clin Transl Med. 2022 Apr 7;12(4):e687. doi: 10.1002/ctm2.687 (PMC8989270; doi:10.1002/ctm2.687)
Supplement: Supplementary file 1 — Supporting Information [file CTM2-12-e687-s001.docx]

**Supplementary Material**

**Supplementary materials and methods**

**1. Animals and DEHP treatment**

C57BL/6 mice were housed under a 12 h light/dark cycle and provided with *ad-libitum* water and food. Female mice were mated with male mice at 16:00 h. Female mice with vaginal plugs were identified the next morning and the day was considered as 0.5 dpc. For reagent preparation, DEHP (Sigma, 36735-1G, Saint Louis, MO, USA) was diluted at a concentration of 40 mg/ml, stored at 4 ℃, and diluted to 0.04 mg/ml with normal saline before use. For DEHP treatment, 40 μg/kg body weight (BW) DEHP was orally administered to pregnant mice starting at 6.5 dpc every day until sample collection. Mice given a corresponding volume of normal saline were regarded as the control groups. All experiments were approved by the Ethics Committee of Qingdao Agriculture University.

**2. Sample collection**

The method for sample collection has been previously described.^1^ Briefly, pregnant mice at 12.5 dpc or 14.5 dpc in DEHP-treatment groups and control groups were euthanized by cervical dislocation. Then female fetuses were separated for gonadal ridge collection. An adequate number of gonadal ridges were washed with normal saline three times and digested with trypsin at 37 ℃ for 3 min to obtain a single cell suspension. The suspension was washed with PBS containing 0.04% bovine serum album (BSA, Solarbio, A8020, Beijing, China) three times and filtered using a 40-μm cell strainer (BD Biosciences 352340, San Jose, USA). Trypan Blue staining was used to ensure that cell viability exceeded 80%.

**3. scRNA-seq and expression matrix generation**

We utilized a Chromium 10× Single Cell System (10× Genomics) to obtain Gel Bead-in-Emulsions (GEMs). Reverse transcription, barcoding, and library establishment were performed using the 10× Genomics Chromium Single Cell 3′ Library and Gel Bead Kit v3, following the manufacturer’s protocol. Paired-end sequencing was conducted via an Illumina HiSeq X Ten sequencer (Illumina, San Diego, USA) at Novogene Bioinformatics Technology Co., Ltd., Beijing, China.

Raw data were then analyzed using CellRanger software v3.1.5 (https://www.10xgenomics.com/) and gene expression data was obtained. Sequencing reports of four different samples were obtained; the essential information of these reports is listed in Figure S1A.

**4. Analysis of scRNA-seq data**

To remove possible doublets, we used the “DoubletFinder” package to filter any abnormal data.^2^ The “Seurat” package was used for normalization, integration, and dimensionality reduction.^3^ We used the *RunUMAP* function to obtain several clusters according to the differential expressions of cells and identified them according to specific markers. Then we extracted the germ cells for detailed analysis. Germ cells from different stages were identified and DEGs at each stage were calculated between DEHP-treatment and control groups. We utilized Metascape (https://metascape.org/gp/index.html) to conduct GO enrichment analysis.

To confirm the developmental trajectory of the germ cells, we conducted RNA velocity analysis via “scVelo”.^4^ In addition, the “FGNet” package and the Cytoscape v3.8.2 plugin GeneMANIA (http://genemania.org/) were also used to construct functional networks of the genes involved in shared GO terms.^5,6^ First, DEGs common to three stages were annotated by topGO, and information about shared GO terms of DEGs was obtained by FGNet. The genes and their annotation information were then loaded into Cytoscape. The interactions between genes were analyzed by the plugin GeneMANIA and functional gene networks were constructed.

**5. Immunofluorescence**

Gonadal ridges were isolated and fixed in paraformaldehyde (PFA) at 4 ℃ overnight. Then gonadal ridges were washed with flowing water, dehydrated, and embedded in paraffin. A rotary microtome (Leica, RM2235, Wetzler, Germany) was used to cut 5 μm thick sections. The slides were deparaffinized and placed in 0.01 M sodium citrate solution at 96 ℃ for 10 min for antigen retrieval. After cooling down to room temperature, slides were blocked with blocking buffer (0.05 M TBS supplemented with 3% BSA and 10% goat serum) for 30 min and incubated with primary antibody at 4 ℃ overnight. The primary antibodies used were: anti-TOMM20 antibody (Abcam, ab78547, Cambridge, UK), anti-phospho-ATM (Ser1981) antibody (Beyotime, AA866, Shanghai, China), anti-phospho-PRKAA1/PRKAA2 (Thr183/Thr172) antibody (Sangon Biotech, D151212, Shanghai, China), anti-DDX4 antibody (Abcam, ab27591), and anti-phospho-ULK1 (Ser555) antibody (ABclonal, AP0760, Wuhan, China). After washing with TBST (0.05 M TBS with 0.1% Tween 20), slides were incubated with Alexa Fluor^®^ 488 goat anti-mouse (Abcam, ab150113) and Alexa Fluor^®^ 555 donkey anti-rabbit (Abcam, ab150074) at 37 ℃ for 45 min. After TBST washing and nuclei staining with Hoechst 33342, the slides were mounted using antifade mounting medium (Boster, AR1109, Wuhan, China) and images were taken using a fluorescence microscope (Olympus, BX51, Tokyo, Japan) or LSCM (Leica, TCS SP5 II) for further analysis using Image J 1.53c.

**6. Chromosome spread**

Gonadal ridges were separated from embryos, washed three times using 0.01 M PBS, and placed in hypo-extraction buffer (30 mM Tris, 50 mM sucrose, 17 mM citric acid, 5 mM EDTA, 2.5 mM DTT, 1 mM PMSF) for 2 h at room temperature. Gonadal ridges were then mechanically shredded in a drop of 0.1 M sucrose on each slide. Subsequently, 500 μl of 1% PFA was added to each slide to fix cells overnight at room temperature. The slides were then air-dried and washed with 0.04% Photo-Flo and 0.05 M TBS. Blocking took place using blocking buffer (4 ml 0.05 M TBS supplemented with 40 μl goat serum, 12 mg BSA and 0.2 μl Triton X-100) for 30 min at room temperature and slides were incubated with anti-SYCP3 (Abcam, ab97672), anti-RAD51 (Abcam, ab133534), or anti-BRCA1 (Boster, PB9015) antibodies for 8 h at 37 ℃. After washing with TBS, the slides were blocked again overnight at 4 ℃ and incubated with the second antibody for 2 h at 37 ℃. After further washing with TBS and nuclei staining with Hoechst 33342, the slides were mounted using antifade mounting medium and captured under a BX51 fluorescence microscope for statistical analysis.

**7. Statistical Analysis**

The data are presented as mean ± SD and no less than three replicates were performed in each experiment. Statistical analysis was conducted using the unpaired t-test in GraphPad Prism v.8.0.2 and SPSS v.21.0. * and ** indicate p < 0.05 and p < 0.01, respectively.

**Reference**

1. Ge W, Wang JJ, Zhang RQ, et al. Dissecting the initiation of female meiosis in the mouse at single-ce

ll resolution. *Cell Mol Life Sci.* 2021;78(2):695-713.

2. McGinnis CS, Murrow LM, Gartner ZJ. DoubletFinder: Doublet Detection in Single-Cell RNA Sequencing Data Using Artificial Nearest Neighbors. *Cell Syst.* 2019;8(4):329-337 e324.

3. Butler A, Hoffman P, Smibert P, Papalexi E, Satija R. Integrating single-cell transcriptomic data across different conditions, technologies, and species. *Nat Biotechnol.* 2018;36(5):411-420.

4. Bergen V, Lange M, Peidli S, Wolf FA, Theis FJ. Generalizing RNA velocity to transient cell states through dynamical modeling. *Nat Biotechnol.* 2020;38(12):1408-1414.

5. Aibar S, Fontanillo C, Droste C, De Las Rivas J. Functional Gene Networks: R/Bioc package to generate and analyse gene networks derived from functional enrichment and clustering. *Bioinformatics.* 2015;31(10):1686-1688.

6. Warde-Farley D, Donaldson SL, Comes O, et al. The GeneMANIA prediction server: biological network integration for gene prioritization and predicting gene function. *Nucleic Acids Res.* 2010;38(Web Server issue):W214-220.

**Supplementary figures**

**
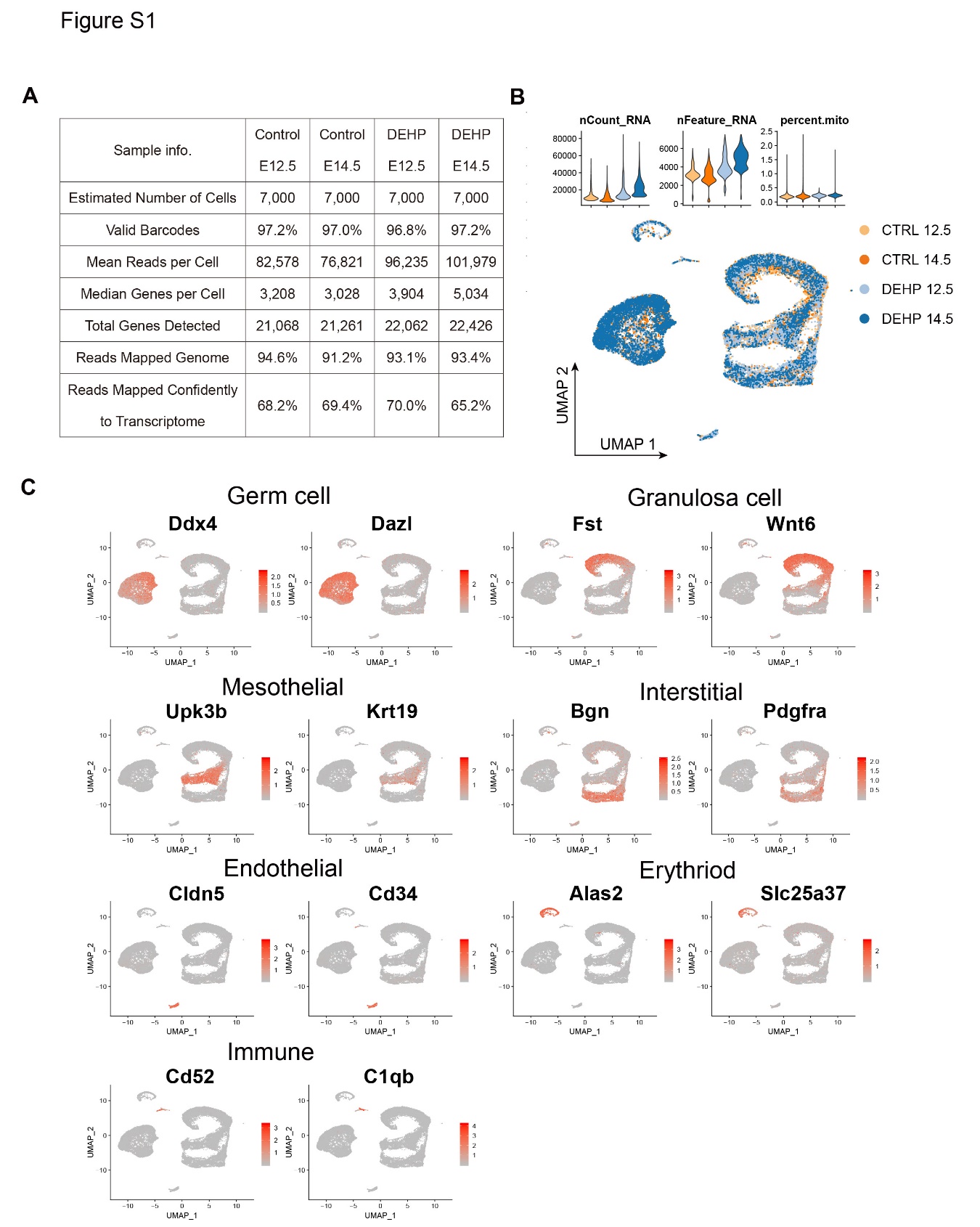
**

**Figure S1. Identification of gonadal ridge cells.** (A) Critical paraments of the sequencing data of four samples. (B) Violin plots of the varieties, counts of mRNA, and percentages of mitochondria mRNA from the four samples (above), and UMAP plot of gonadal ridge cells colored by four samples (below) (C) Expression plots of some marker genes of different gonadal cell types.


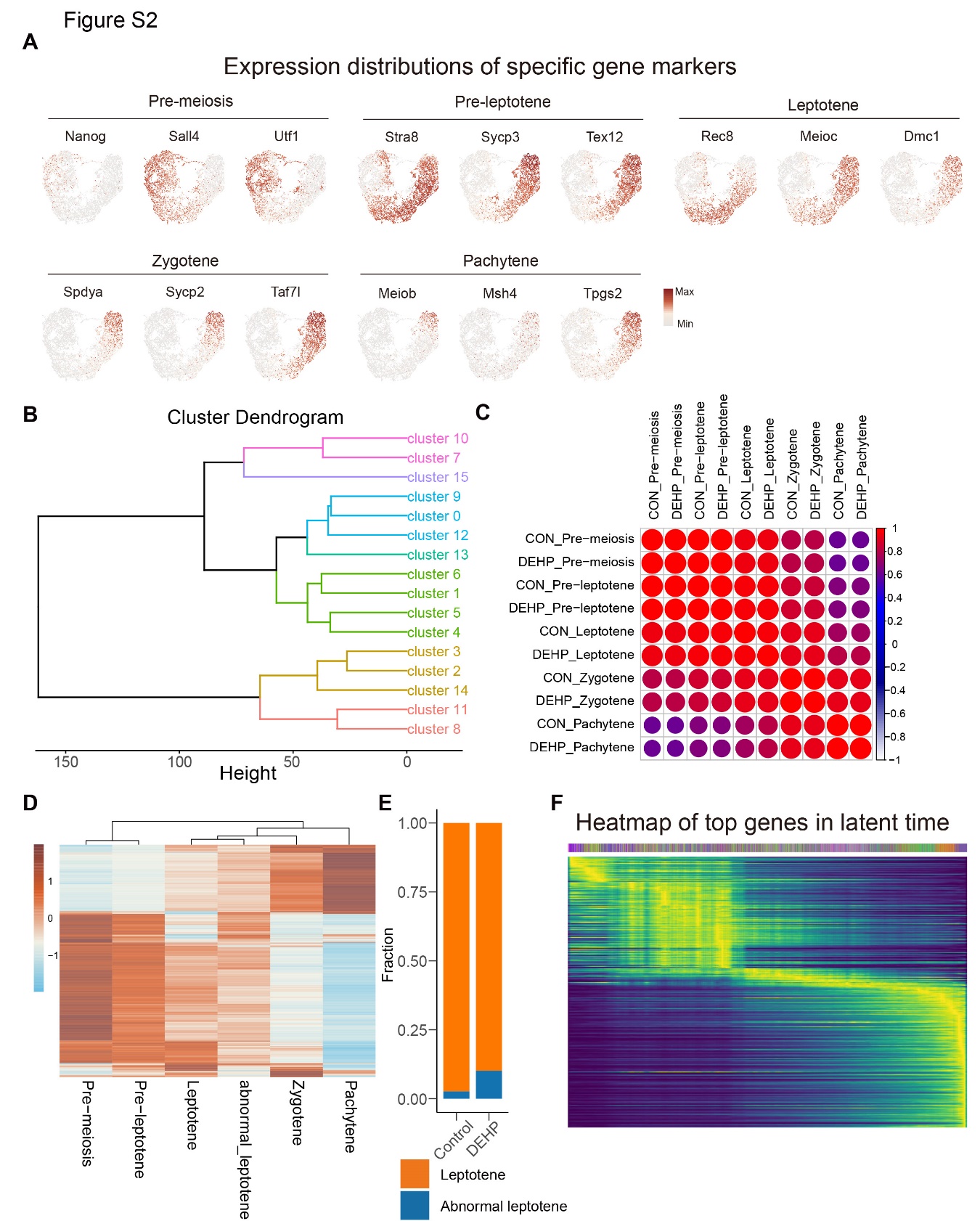


**Figure S2. Identification of germ cells.** (A) Expression plots of some marker genes of different developmental stages of germ cells. (B) Dendrogram plot of 16 different germ cell clusters. (C) Correlation plots of the different identified germ cell stages. (D) Heatmap of the different identified germ cell stages. (E) Stacked bar plot of the proportion of abnormal leptotene cells and normal leptotene cells in the Control and DEHP groups. (F) Heatmap of top genes in germ cells arranged by velocity pseudotime.


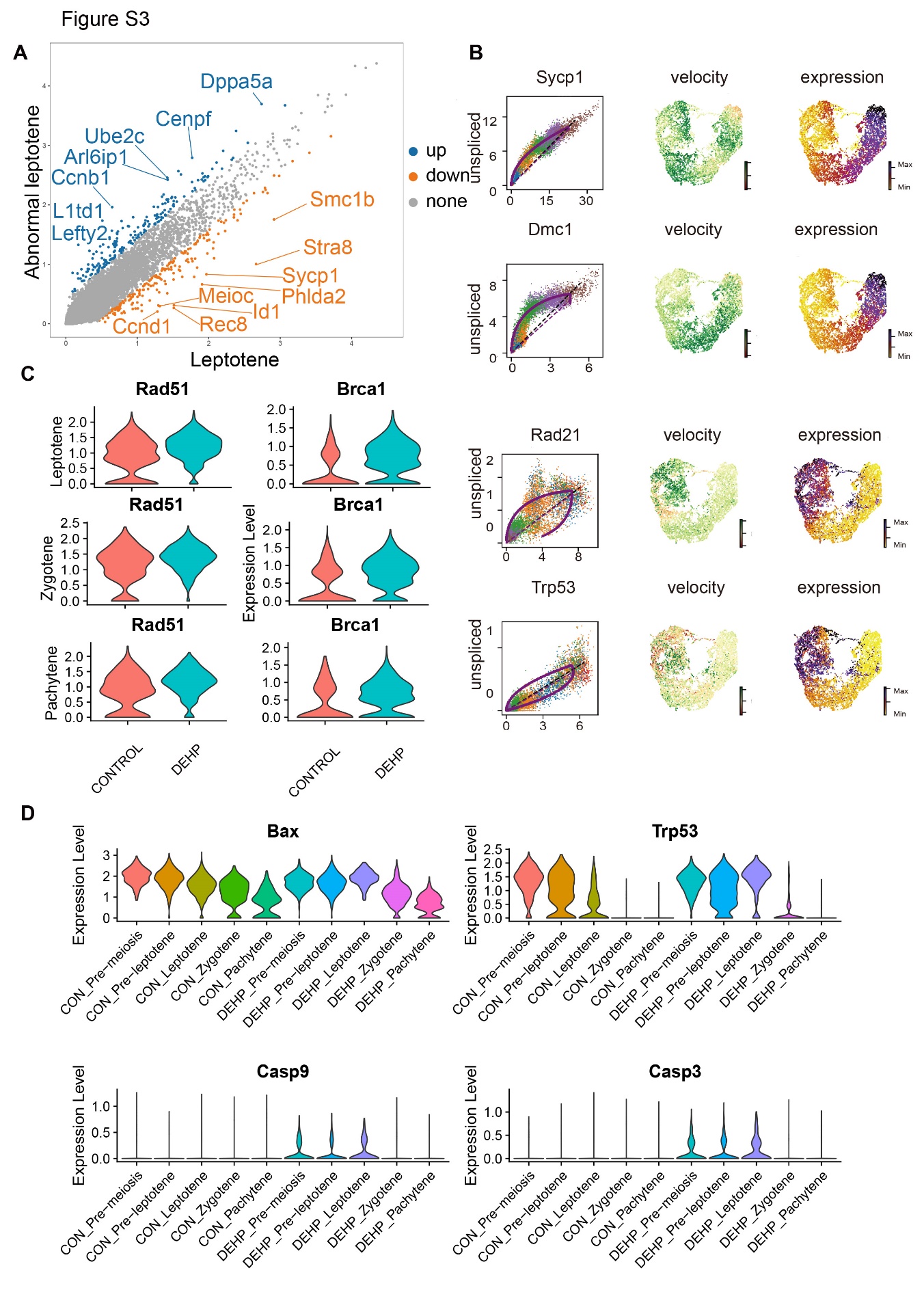


**Figure S3. Influence of DEHP on germ cells.** (A) Gene expression differences between leptotene and abnormal leptotene germ cells. The x- and y-axis indicate the normalized expression level. Blue dots represent the upregulated genes in the abnormal leptotene germ cells. Orange dots represent the downregulated genes in the abnormal leptotene germ cells. Grey dots represent the rest genes. (B) The spliced vs. unspliced mRNA plot, velocity plot, and expression plot of some genes. Black lines in the first plot indicate the estimated ‘steady-state’ ratio, which corresponds to a constant transcriptional state. (C) Violin plots of *Rad51* and *Brca1* in leptotene, zygotene, and pachytene stages of germ cells from the control and DEHP-treated groups. (D) Violin plots of the apoptosis-related genes in all the identified stages of germ cells.


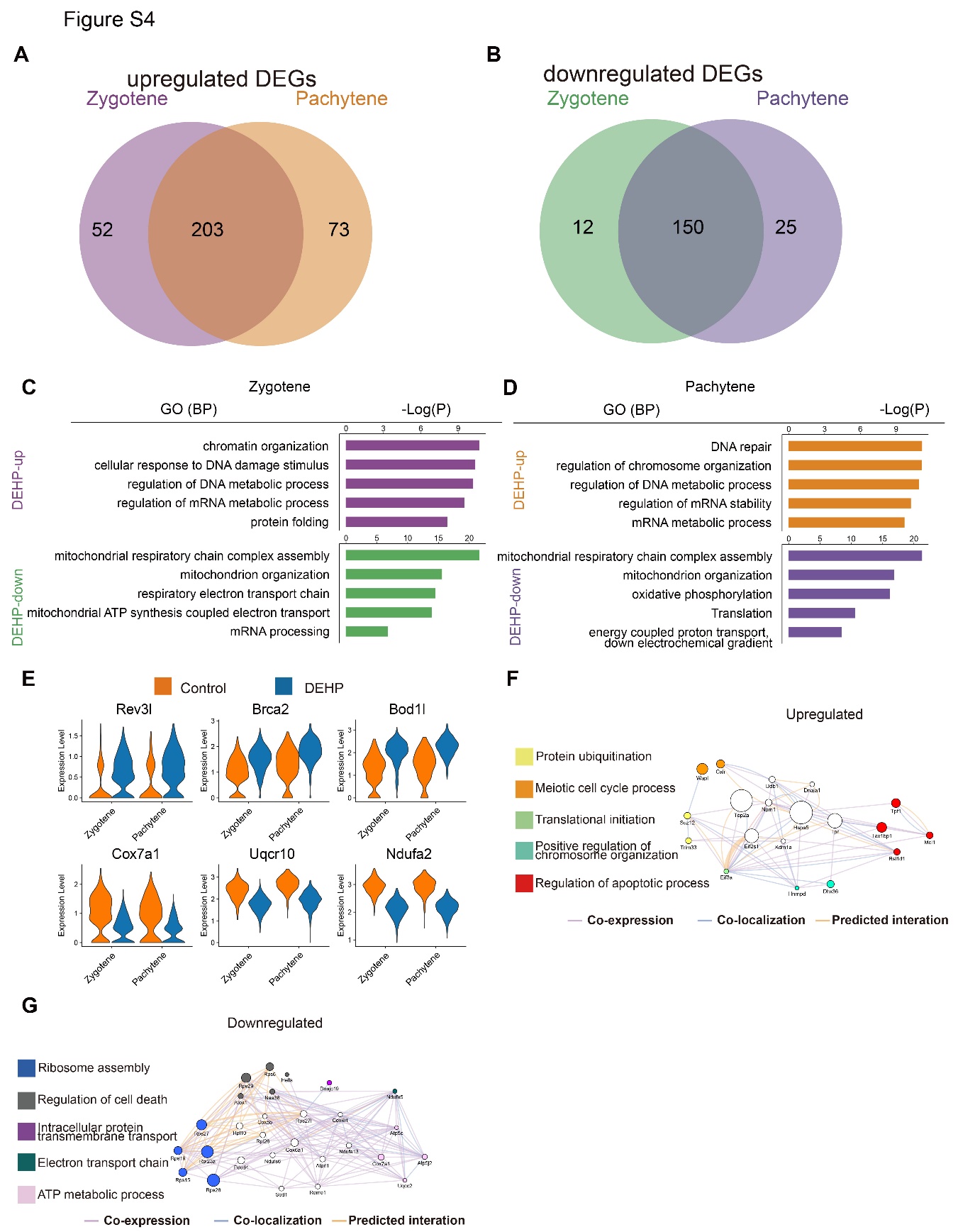


**Figure S4. Analysis of different stages and treatment groups of germ cells** (A-B) Venn plots of upregulated (A) and downregulated (B) DEGs between the control and DEHP-treated groups of zygotene and pachytene cells. (C-D) GO term enrichment analysis of upregulated (top) and downregulated (bottom) DEGs in the DEHP-treated cells during zygotene (C) and pachytene (D) stages. (E) Violin plots of expressions of some genes between control and DEHP-treated cells in the zygotene and pachytene stage. (F-G) Functional gene networks of upregulated (F) and downregulated (G) DEGs in all of the three stages and their interaction relationships. Similar colored circles indicate genes belonging to the same GO term and white circles represent genes belonging to more than one GO term. The size of the circles represents the $\text{log}_{\text{2}}$(foldchange) between the control and DEHP-treated groups.


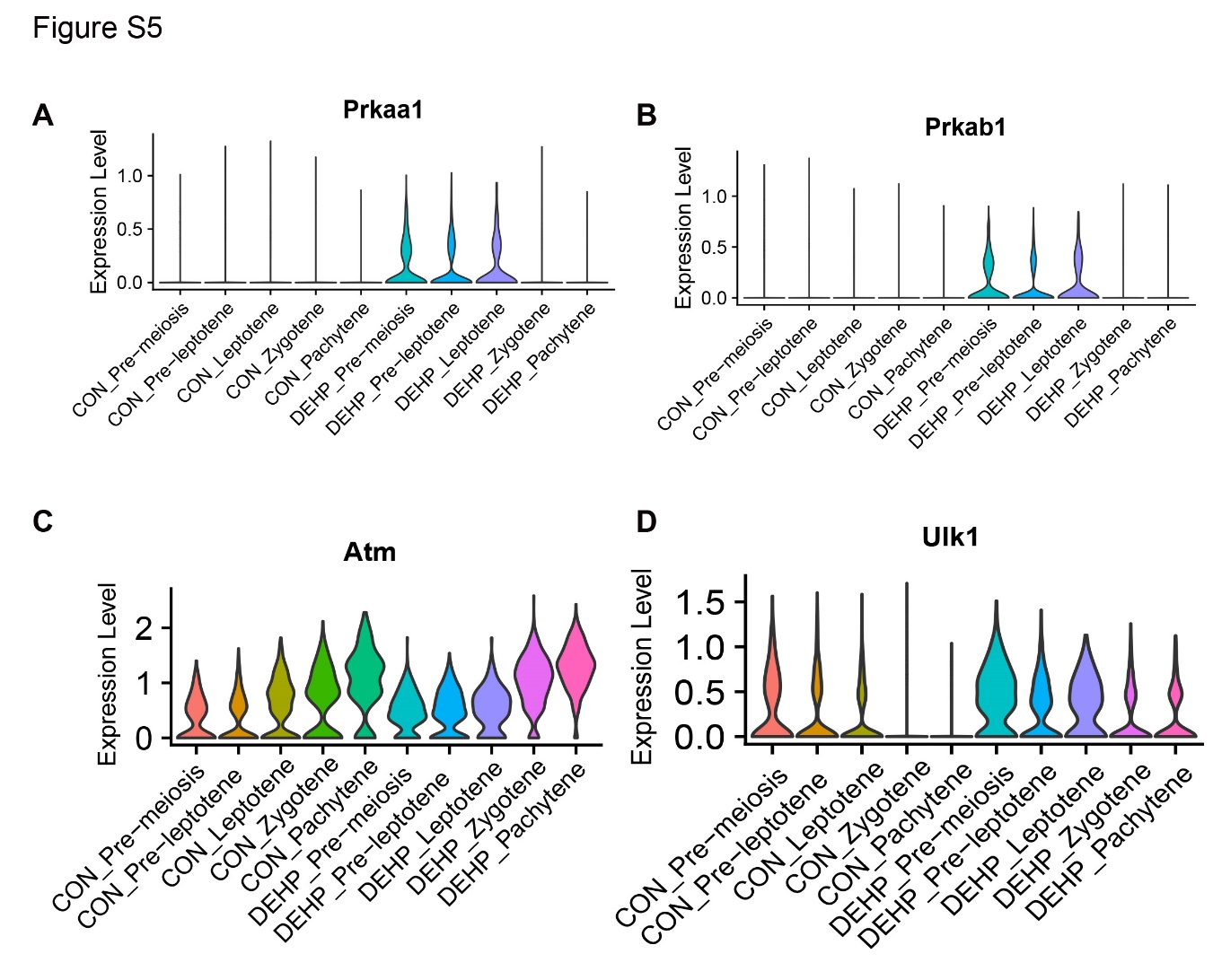


**Figure S5. The expression of some important genes altered by DEHP.** (A) Violin plots of the AMPK subunit *Prkaa1* in all the identified stages of germ cells. (B) Violin plots of the AMPK subunit *Prkab1* in all the identified stages of germ cells. (C) Violin plots of *Atm* in all the identified stages of germ cells. (D) Violin plots of *Ulk1* in all the identified stages of germ cells.


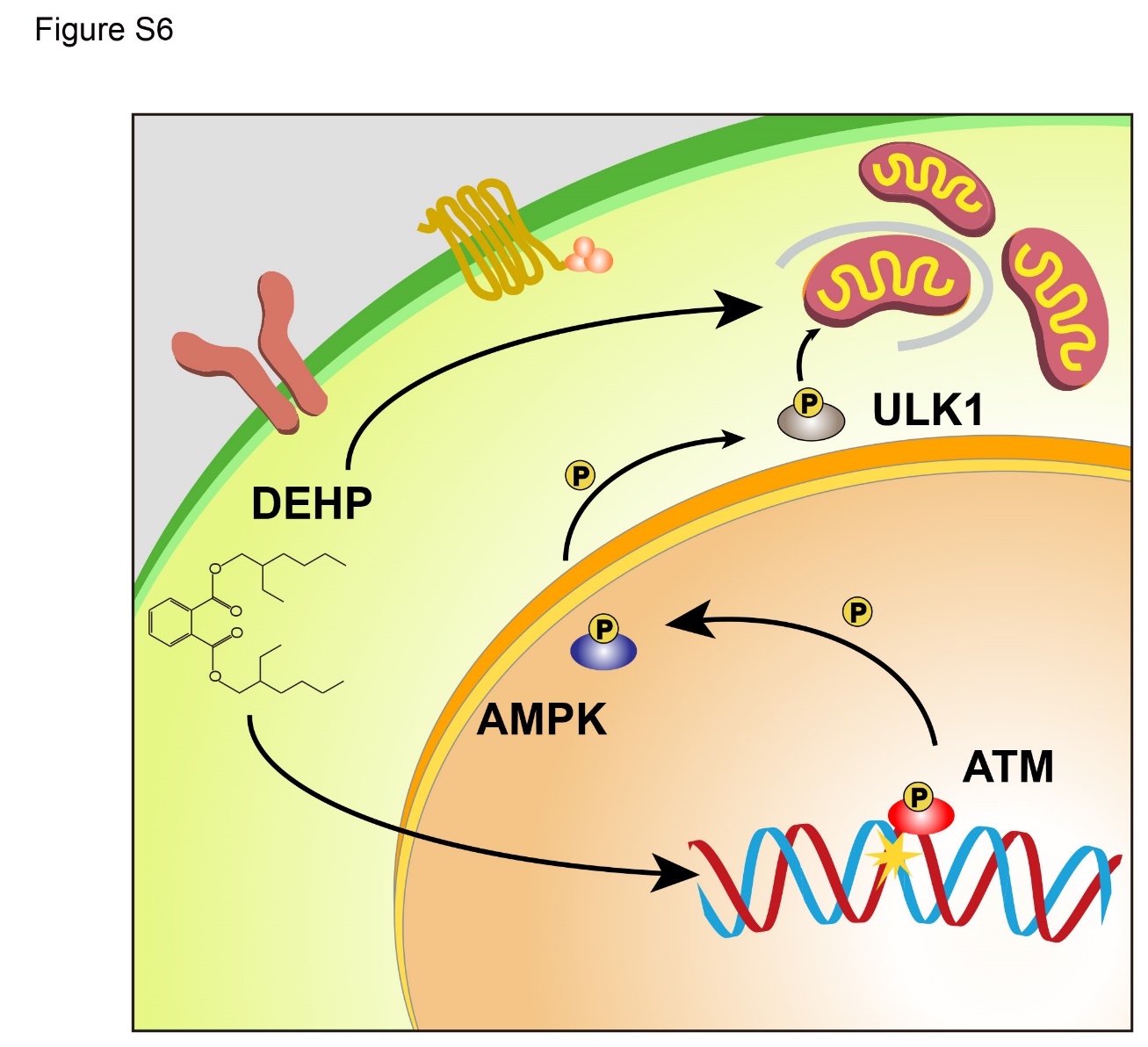


**Figure S6. Diagram of the possible pathway by which DEHP affected germ cells by inducing phosphorylated AMPK.**
